# Laboratory-based Surveillance for Hepatitis E Virus Infection, United States, 2005–2012

**DOI:** 10.3201/eid1902.120961

**Published:** 2013-02

**Authors:** Jan Drobeniuc, Tracy Greene-Montfort, Ngoc-Thao Le, Tonya R. Mixson-Hayden, Lilia Ganova-Raeva, Chen Dong, Ryan T. Novak, Umid M. Sharapov, Rania A. Tohme, Eyasu Teshale, Saleem Kamili, Chong-Gee Teo

**Affiliations:** Author affiliation: Centers for Disease Control and Prevention, Atlanta, GA, USA

**Keywords:** jaundice, incidence, infectious disease transmission, travel, immunocompromised host, diagnostic techniques, hepatitis E, hepatitis E virus, HEV, Unites States, differential diagnosis, travel-related infections, surveillance, viruses

## Abstract

Clinicians should consider this virus in the differential diagnosis of hepatitis, regardless of patient travel history.

## Medscape CME Quiz

## Earning CME Credit

To obtain credit, you should first read the journal article. After reading the article, you should be able to answer the following, related, multiple-choice questions. To complete the questions (with a minimum 70% passing score) and earn continuing medical education (CME) credit, please go to www.medscape.org/journal/eid. Credit cannot be obtained for tests completed on paper, although you may use the worksheet below to keep a record of your answers. You must be a registered user on Medscape.org. If you are not registered on Medscape.org, please click on the New Users: Free Registration link on the left hand side of the website to register. Only one answer is correct for each question. Once you successfully answer all post-test questions you will be able to view and/or print your certificate. For questions regarding the content of this activity, contact the accredited provider, CME@medscape.net. For technical assistance, contact CME@webmd.net. American Medical Association’s Physician’s Recognition Award (AMA PRA) credits are accepted in the US as evidence of participation in CME activities. For further information on this award, please refer to http://www.ama-assn.org/ama/pub/category/2922.html. The AMA has determined that physicians not licensed in the US who participate in this CME activity are eligible for *AMA PRA Category 1 Credits*™. Through agreements that the AMA has made with agencies in some countries, AMA PRA credit may be acceptable as evidence of participation in CME activities. If you are not licensed in the US, please complete the questions online, print the certificate and present it to your national medical association for review.

## Article Title:  Laboratory-based Surveillance for Hepatitis E Virus Infection, United States, 2005–2012

## CME Questions

**1. You are a consultant advising an HMO regarding the percentage of hepatitis E among US patients with hepatitis. Based on the study by Dr. Drobeniuc and colleagues, which of the following statements would most likely appear in your report?**

A. Hepatitis E was present in more than half of patients who were seronegative for acute hepatitis A and B

B. Among patients with hepatitis E, only one quarter had recently traveled abroad

C. Among patients with hepatitis E, half the patients had acute and half the patients had chronic hepatitis

D. Hepatitis E virus (HEV) infection was determined by testing for IgM and IgG anti-HEV and for HEV RNA

**2. Based on the study by Dr. Drobeniuc and colleagues, which of the following statements about group characteristics of nontravelers vs travelers with hepatitis E is most likely correct?**

A. Nontravelers were older than travelers 

B. Nontravelers were more likely than travelers to be jaundiced

C. Nontravelers comprised fewer South Asians than travelers 

D. Nontravelers were less likely than travelers to be solid organ transplant recipients

**3. Based on the study by Dr. Drobeniuc and colleagues, which of the following statements about HEV genotypes among nontravelers vs travelers with hepatitis E is most likely correct?**

A. Nontravelers were infected exclusively by HEV genotype 1 strains

B. Nontravelers were infected by HEV genotype 3 and 4 strains

C. Travelers were infected exclusively by HEV genotype 3 strains

D. The findings suggest that the nontravelers were infected by HEV that was circulating autochthonously in the United States.

## Activity Evaluation

**Table:** 

**1. The activity supported the learning objectives.**				
Strongly Disagree				Strongly Agree
1	2	3	4	5
**2. The material was organized clearly for learning to occur.**				
Strongly Disagree				Strongly Agree
1	2	3	4	5
**3. The content learned from this activity will impact my practice.**				
Strongly Disagree				Strongly Agree
1	2	3	4	5
**4. The activity was presented objectively and free of commercial bias.**				
Strongly Disagree				Strongly Agree
1	2	3	4	5
