# Supplementary material for: Laboratory-based Surveillance for Hepatitis E Virus Infection, United States, 2005–2012
Source: Emerg Infect Dis. 2013 Feb;19(2):218–22. doi: 10.3201/eid1902.120961 (PMC3563276; doi:10.3201/eid1902.120961)
Supplement: Related texts

# Research article: Laboratory-based Surveillance for Hepatitis E Virus Infection, United States, 2005–2012

**Authors:** Jan Drobeniuc, Tracy Greene-Montfort, Ngoc-Thao Le, Tonya R. Mixson-Hayden, Lilia Ganova-Raeva, Chen Dong, Ryan T. Novak, Umid M. Sharapov, Rania A. Tohme, Eyasu Teshale, Saleem Kamili, Chong-Gee Teo

**Affiliations:** Author affiliation: Centers for Disease Control and Prevention, Atlanta, GA, USA

## Abstract

To investigate characteristics of hepatitis E cases in the United States, we tested samples from persons seronegative for acute hepatitis A and B whose clinical specimens were referred to the Centers for Disease Control and Prevention during June 2005–March 2012 for hepatitis E virus (HEV) testing. We found that 26 (17%) of 154 persons tested had hepatitis E. Of these, 15 had not recently traveled abroad (nontravelers), and 11 had (travelers). Compared with travelers, nontravelers were older (median 61 vs. 32 years of age) and more likely to be anicteric (53% vs. 8%); the nontraveler group also had fewer persons of South Asian ethnicity (7% vs. 73%) and more solid-organ transplant recipients (47% vs. 0). HEV genotype 3 was characterized from 8 nontravelers and genotypes 1 or 4 from 4 travelers. Clinicians should consider HEV infection in the differential diagnosis of hepatitis, regardless of patient travel history.

Hepatitis E in Africa, southern and central Asia, and Central America causes occasional outbreaks of jaundice and, between outbreaks, occurrences of sporadic jaundice. Primarily spread by waterborne transmission, the disease tends to resolve spontaneously, although fulminant hepatic failure can ensue (*1*). In eastern Asia and Europe, sporadic hepatitis E, whether imported after return from international travel or acquired indigenously, has been observed; the indigenous form is thought to be foodborne (*2*). Although the disease is largely self-limiting, in Europe, chronic hepatitis E, which may lead to cirrhosis, is increasingly recognized among solid-organ transplant recipients (SOTRs) (*3*). 

The causative agent of hepatitis E is hepatitis E virus (HEV), of which 4 genotypes are found in humans. Genotypes 1 and 2 circulate in regions where waterborne transmission is common; genotype 3 is prevalent in eastern Asia and the West and genotype 4 in eastern Asia. Genotypes 1 and 2 infect humans, but genotypes 3 and 4 infect humans and animals, predominantly pigs (*4*).

In the United States, HEV imported into the country after travel to regions to which waterborne HEV transmission is endemic is well recognized (*5*–*7*). Recently, 21% of participants of the US-based Third National Health and Nutrition Examination Survey were found seropositive for IgG against HEV (*8*). This unexpectedly high prevalence rate would not be ascribable to imported HEV infection alone. Indeed, cases of hepatitis E unassociated with travel abroad have been observed in the United States, implying infection by indigenous HEV strains (*9*–*14*). Moreover, the increasing number of reports from Europe of hepatitis E among SOTRs (*3*,*15*) suggests that SOTRS in the United States might be similarly susceptible to the disease.

We report a study of demographic, clinical, travel-related, and virologic characteristics of persons with hepatitis E derived from a diverse patient base. Critical to this investigation was the application of a validated serologic assay for detecting IgM against HEV (*16*), the marker of recent HEV infection, as well as a real-time reverse transcription PCR (RT-PCR) that had been validated to detect, to high sensitivity, HEV RNA (*17*), which is an indicator of active HEV shedding. Together, these 2 assays enabled us to identify patients with incident hepatitis E.

## Methods

### Samples and Patients

The Centers for Disease Control and Prevention (CDC) conducts HEV testing of serum and stool samples referred by health care providers, public health departments, and diagnostic laboratories in the United States (*18*). Referrers are requested to fill out a standardized questionnaire of patients’ demographics, clinical and laboratory test features, and risks for HEV infection, including recent international travel and destinations visited; the completed questionnaire is submitted along with the test specimens (*18*). Persons whose specimens were received during June 2005–March 2012 and reported as being negative for IgM against hepatitis A virus and hepatitis B core antigen, regardless of positivity for IgG against hepatitis C virus, were considered for inclusion into the study.

### Assays

An earlier, pangenotypic evaluation by CDC of 6 serologic assays for IgM against HEV identified the assay manufactured by Diagnostic Systems (Saronno, Italy) as having the best performance characteristics (*16*). Its diagnostic sensitivity and specificity were 98% and 95.2%, respectively, and its analytic sensitivity was 9 Walter Reed Units/mL. For this study, the assay was used to detect IgM against HEV in test samples. IgG against HEV was tested by applying an assay from the same manufacturer. Serum and stool samples were tested for HEV RNA by a real-time RT-PCR, capable of detecting HEV genotypes 1–4 to a sensitivity of 4 HEV genome-equivalents/mL, to amplify a 69-bp fragment in open reading frame (ORF) 3 of the HEV genome (*19*). Application of that assay enabled our laboratory to attain perfect detection scores in a recent international evaluation of 20 laboratories conducting HEV RNA testing (*17*). Samples found to be positive for HEV RNA were subjected to another RT-PCR to generate amplicons from a 258-bp segment from ORF1, which were then processed for nucleotide sequencing and phylogenetic analyses (*20*).

### Statistics

Distributions of variables were assessed by using the Kruskal-Wallis test and the χ^2^ test with the Yates correction or the Fisher exact test, as appropriate. Univariate and bivariate data analyses were conducted by using Epi Info (wwwn.cdc.gov/EpiInfo/html/prevVersion.htm). 

### Case Definition 

A case of hepatitis E was defined as illness in a person in whom IgM and IgG against HEV in serum or HEV RNA in serum or stool samples were detected. A person in whom IgM but not IgG against HEV was detected in serum was excluded unless HEV RNA was found or IgG againstHEV was detected in follow-up serum samples. A person in whom IgG but not IgM against HEV was detected in serum samples was included if HEV RNA was found in serum or stool samples.

## Results

Of 154 persons whose specimens fulfilled the inclusion criteria, 26 (17%) met the case definition for hepatitis E. Case-patients were between 14–67 years of age (median 43 years); 19 (73%) were male. Fifteen (58%) were white, 9 (36%) South Asian, and 2 (8%) Hispanic. None were seropositive for IgG against HEV. Eighteen (69%) case-patients were jaundiced, and 7 (27%) were SOTRs, the allografts received being kidney (3), liver (2), kidney and pancreas (1), and heart and lungs (1). Fifteen case-patients (58%) who reported not having traveled outside the United States in the previous 2 months were classified as nontravelers; the remaining 11, who had traveled abroad, were classified as travelers. 

The Table summarizes the demographic, clinical, and virologic data for individual case-patients. Compared with travelers, nontravelers were older (median age 61 vs. 32 years of age; p<0.05) and more likely to be anicteric (not jaundiced; 8/15 [53%] vs. 1/11 [8%]; p = 0.02). The nontraveler group also included fewer South Asians (1/15 [7%] vs. 8/11 [73%]; p<0.001) and more SOTRs (7/15 [47%] vs. 0; p = 0.02). Differences in sex distribution were not significant.

**Table T-2-1:** Demographic, clinical, travel-related, and virologic characteristics for patients with hepatitis E, United States, 2005–2012*

Travel history and case-patient no.	Age, y/sex	Race/ ethnicity	State of residence	Transplant (organ)	Jaundice	Countries visited	Anti-HEV SCR		HEV genotype	HEV viral load†
							IgM	IgG		
No recent international travel‡										
NT1	61/M	White	FL	No	Yes	NA	7.5	5.7	3	NA
NT2	45/M	White	CA	No	Yes	NA	3.7	4	–	–
NT3	63/M	White	SD	Yes (kidney)	No	NA	7.2	5.4	3	NA
NT4	61/M	South Asian	IL	Yes (liver)	No	NA	1.9	5.9	3	NA
NT5	67/M	White	FL	No	Yes	NA	6.3	1.3	–	–
NT6	44/F	Hispanic	TX	No	Yes§	NA	3.1	3.7	3	NA
NT7	21/F	Hispanic	TX	No	Yes¶	NA	2.2	1.6	–	–
NT8	67/M	White	IL	Yes (heart and lungs)	Yes	NA	3	3.3	–	–
NT9	42/M	White	WI	No	Yes	NA	6	6.6	–	–
NT10	62/F	White	IL	Yes (kidney)	No	NA	2.9	8.9	–	–
NT11	26/M	White	PA	Yes (kidney)	No	NA	5.3	8.3	3	7.8 × 10^2^
NT12	40/F	White	NY	Yes (kidney and pancreas)	No#	NA	7.7	12.9	3	1.4 × 10^3^
NT13	64/M	White	CT	Yes (liver)	Yes	NA	9.2	1.3	3	1.4 × 10^4^
NT14	29/F	White	MI	No	No**	NA	6.6	9.8	–	–
NT15	62/M	White	NY	No	No	NA	Neg	9.6	3	1.5 × 10^3^
Recent international travel‡										
T1	35/M	South Asian	DE	No	Yes	India	2.3	4.5	1	1.8 × 10^2^
T2	14/F	South Asian	TX	No	Yes	India	7.3	5.8	–	–
T3	32/F	South Asian	TX	No	Yes	India	3.7	5.8	–	–
T4	24/M	South Asian	TX	No	Yes	India	2.3	2	–	–
T5	35/M	White	IL	No	No	India and Indonesia	2.9	8.9	–	–
T6	24/M	White	MD	No	Yes	Afghanistan and Dubai	6.9	9.4	–	–
T7	63/M	White	AL	No	Yes	China	7.9	Neg	4	2.4 × 10^2^
T8	23/M	South Asian	ME	No­­	Yes	Bangladesh	7.6	10.8	–	–
T9	53/M	South Asian	MD	No	Yes††	India	9.2	9.4	–	–
T10	66/M	South Asian	TX	No	Yes	India	5.5	11.7	1	1.8 × 10^2^
T11	22/M	South Asian	MD	No	Yes	India	9.9	10.9	1	8.3 × 10^5^

*HEV, hepatitis E virus; SCR, signal/cutoff ratio; NT, nontraveler; NA, not applicable (quantitative reverse transcription PCR not done); –, HEV not detected or genotype not tested; Neg, negative. †In genome-equivalents/mL. ‡Within 2 mo before illness or visit to physician. §Fulminant hepatic failure developed but resolved (*14*). ¶Fulminant hepatic failure developed; patient died at time of liver transplantation (*14*). #Initial illness was meningitis. **Asymptomatic; tested for HEV because of recent miscarriage. ††Fulminant hepatic failure developed, requiring liver transplantation; patient survived.

Three case-patients (NT6, NT7, and T9; Tabl[Table T-2-1]e) were documented to have fulminant hepatic failure. Two of them required liver transplantation; 1 died and 1 survived.

HEV RNA was amplified from 12 case-patients (46%). The rate of HEV RNA detection among SOTRs (5/7; 71%) was higher than that among non-SOTRs (7/19; 37%), but this difference was not significant. HEV genotype 1 was characterized from 3 travelers, genotype 3 from 8 nontravelers (including the 5 SOTRs), and genotype 4 from 1 traveler. The Figure displays the genetic diversity of HEV carried.

**Figure:** Genetic relatedness among hepatitis E virus (HEV) strains identified in hepatitis E cases, United States. Phylogenetic tree was constructed from a segment of HEV open reading frame 1 generated in MEGA5 (www.megasoftware.net) by using the neighbor-joining method. Country, year reported, and numeric or GenBank accession number assignment are denoted. Scale bar indicates genetic distance.

## Discussion

We identified 26 case-patients with hepatitis E in the United States. No distinction was made between acute and chronic hepatitis E. Whereas acute hepatitis among non-SOTRs was readily identifiable (most were jaundiced when test specimens were drawn), it was difficult to asses whether disease in SOTRs was at the acute or chronic stage during specimen sampling, because positivity for IgM against HEV or HEV RNA could reflect either stage (*3*). Thus, the case definition was kept broad to identify both stages of disease. As the study was not primarily prospective, the natural history of hepatitis E among the case-patients was largely unknown. Nevertheless, adverse outcomes could be documented for 3 case-patients, in whom fulminant hepatic failure developed.

Hepatitis E cases were found among persons who had not recently traveled abroad and those who had. Nontravelers tended to be older than travelers, a trend consistent with the finding recently reported by the Drug-Induced Liver Injury Network of 9 patients seropositive for IgM against HEV whose mean age was 67 years (*21*) and with similar observations in Japan and Europe (*1*,*2*,*22*). The higher proportion of anicteric persons in the nontraveler group reflects its inclusion of all SOTRs, which in Europe have been observed to have largely asymptomatic infections (*3*).

Nontravelers were infected exclusively by HEV genotype 3 strains. These strains clustered with HEV previously found in case-patients with nonimported acute hepatitis E (*9*–*12*,*14*) in the United States (Figure), suggesting that the nontravelers were infected by autochthonously circulating HEV. The similarity between HEV genotype 3 strains identified in nontravelers with those in swine (*4*) (Figure) suggests, but does not prove, HEV transmission linkage between humans and pigs (*2*). Evidence of HEV infection acquired after consumption of inadequately cooked meat and offal originating from pigs, boars, and deer has been reported from Japan (*2*) and France (*23*). Elsewhere, including the United States, evidence implicating non–travel-associated hepatitis E as a zoonosis remains weak (*24*).

The patient base from which hepatitis E cases were identified was nonselective, broad, and derived from multiple health care provider contexts. Nonetheless, data from this study were not generated from an established, systematic program of epidemiologic surveillance. Accordingly, the cases identified here may not fully represent the extent of hepatitis E in the United States. The larger number of cases among nontravelers likely reflects more persons living in the United States who do not travel abroad compared with those who do, and the many SOTRs identified with hepatitis E could be an overrepresentation resulting from increasing awareness among physicians of the predilection of the SOTR patient subpopulation to HEV infection (*3*,*15*). Future surveillance of hepatitis E may need to sample source populations from more diverse settings, such as gastroenterology/hepatology clinics (*21*), travel clinics (*6*), and the military (*7*). We recently reported findings from a study of HEV infection among immunocompromised patients other than SOTRs (*25*).

This study has provided insight into nonimported and imported hepatitis E in the United States. The nonimported form was observed to affect SOTRs, be able to lead to adverse outcomes, and be associated with infection by HEV genotype 3. The extent of nonimported hepatitis E in the United States merits further investigation, as does the role of autochthonous transmission of genotype 3 HEV strains. In clinical practice, entry of hepatitis E into the differential diagnosis of suspected hepatitis, regardless of the patient’s travel history, would be appropriate.

## References

[1] Labrique A, Kuniholm MH, Nelson KE. The global impact of hepatitis E—new horizons for an emerging virus. In: Grayson L, editor. Emerging Infections 9. Washington (DC): American Society for Microbiology; 2010. p. 54–92.

[2] Teo CG. Much meat, much malady: changing perceptions of the epidemiology of hepatitis E. Clin Microbiol Infect. 2010;16:24–32. 10.1111/j.1469-0691.2009.03111.x20002688

[3] Legrand-Abravanel F, Kamar N, Sandres-Saune K, Garrouste C, Dubois M, Mansuy JM, Characteristics of autochthonous hepatitis E virus infection in solid-organ transplant recipients in France. J Infect Dis. 2010;202:835–44. 10.1086/65589920695798

[4] Dong C, Meng J, Dai X, Liang JH, Feagins AR, Meng XJ, Restricted enzooticity of hepatitis E virus genotypes 1 to 4 in the United States. J Clin Microbiol. 2011;49:4164–72. 10.1128/JCM.05481-1121998412PMC3232956

[5] Centers for Disease Control and Prevention. Hepatitis E among U.S. travelers, 1989–1992. MMWR Morb Mortal Wkly Rep. 1993;42:1–4 .8418395

[6] Ooi WW, Gawoski JM, Yarbough PO, Pankey GA. Hepatitis E seroconversion in United States travelers abroad. Am J Trop Med Hyg. 1999;61:822–4 .1058691810.4269/ajtmh.1999.61.822

[7] Eick A, Ticehurst J, Tobler S, Nevin R, Lindler L, Hu Z, Hepatitis E seroprevalence and seroconversion among US military service members deployed to Afghanistan. J Infect Dis. 2010;202:1302–8. 10.1086/65659820863230

[8] Kuniholm MH, Purcell RH, McQuillan GM, Engle RE, Wasley A, Nelson KE. Epidemiology of hepatitis E virus in the United States: results from the Third National Health and Nutrition Examination Survey, 1988–1994. J Infect Dis. 2009;200:48–56. 10.1086/59931919473098PMC2762746

[9] Kwo PY, Schlauder GG, Carpenter HA, Murphy PJ, Rosenblatt JE, Dawson GJ, Acute hepatitis E by a new isolate acquired in the United States. Mayo Clin Proc. 1997;72:1133–6. 10.4065/72.12.11339413292

[10] Erker JC, Desai SM, Schlauder GG, Dawson GJ, Mushahwar IK. A hepatitis E virus variant from the United States: molecular characterization and transmission in cynomolgus macaques. J Gen Virol. 1999;80:681–90 .1009200810.1099/0022-1317-80-3-681

[11] Tsang TH, Denison EK, Williams HV, Venczel LV, Ginsberg MM, Vugia DJ. Acute hepatitis E infection acquired in California. Clin Infect Dis. 2000;30:618–9. 10.1086/31373010722465

[12] Amon JJ, Drobeniuc J, Bower WA, Magaña JC, Escobedo MA, Williams IT, Locally acquired hepatitis E virus infection, El Paso, Texas. J Med Virol. 2006;78:741–6. 10.1002/jmv.2061716628579

[13] Curry JA, Adams N, Crum-Cianflone NF. Acute hepatitis E virus infection in an HIV-infected person in the United States. Ann Intern Med. 2009;150:226–7 .1918992510.7326/0003-4819-150-3-200902030-00028PMC2720044

[14] Tohme RA, Drobeniuc J, Sanches R, Heseltine G, Alsip B, Kamili S, Acute hepatitis associated with autochthonous hepatitis E virus infection — San Antonio, Texas, 2009. Clin Infect Dis. 2011;53:793–6. 10.1093/cid/cir45321896699

[15] Pas SD, de Man RA, Mulders C, Balk AH, van Hal PT, Weimar W, Hepatitis E virus infection among solid organ transplant recipients, the Netherlands. Emerg Infect Dis. 2012;18:869–72. 10.3201/eid1805.11171222516170PMC3358074

[16] Drobeniuc J, Meng J, Reuter G, Greene-Montfort T, Khudyakova N, Dimitrova Z, Serologic assays specific to immunoglobulin M antibodies against hepatitis E virus: pangenotypic evaluation of performances. Clin Infect Dis. 2010;51:e24–7. 10.1086/65480120578874

[17] Baylis SA, Hanschmann KM, Blümel J, Nübling CM; HEV Collaborative Study Group. Standardization of hepatitis E virus (HEV) nucleic acid amplification technique–based assays: an initial study to evaluate a panel of HEV strains and investigate laboratory performance. J Clin Microbiol. 2011;49:1234–9. 10.1128/JCM.02578-1021307208PMC3122834

[18] Centers for Disease Control and Prevention. Hepatitis E information for healthcare professionals [cited 2012 Sep 14]. http://www.cdc.gov/hepatitis/HEV/LabTestingRequests.htm

[19] Jothikumar N, Cromeans TL, Robertson BH, Meng XJ, Hill VR. A broadly reactive one-step real-time RT-PCR assay for rapid and sensitive detection of hepatitis E virus. J Virol Methods. 2006;131:65–71. 10.1016/j.jviromet.2005.07.00416125257

[20] Chatterjee R, Tsarev S, Pillot J, Coursaget P, Emerson SU, Purcell RH. African strains of hepatitis E virus that are distinct from Asian strains. J Med Virol. 1997;53:139–44. 10.1002/(SICI)1096-9071(199710)53:2<139::AID-JMV5>3.0.CO;2-A9334924

[21] Davern TJ, Chalasani N, Fontana RJ, Hayashi PH, Protiva P, Kleiner DE, ; Drug-Induced Liver Injury Network (DILIN). Acute hepatitis E infection accounts for some cases of suspected drug-induced liver injury. Gastroenterology. 2011;141:1665–72.e1–9. 10.1053/j.gastro.2011.07.051PMC365454021855518

[22] Romanò L, Paladini S, Tagliacarne C, Canuti M, Bianchi S, Zanetti AR. Hepatitis E in Italy: a long-term prospective study. J Hepatol. 2011;54:34–40. 10.1016/j.jhep.2010.06.01720888660

[23] Colson P, Borentain P, Queyriaux B, Kaba M, Moal V, Gallian P, Pig liver sausage as a source of hepatitis E virus transmission to humans. J Infect Dis. 2010;202:825–34. 10.1086/65589820695796

[24] Wilhelm BJ, Rajić A, Greig J, Waddell L, Trottier G, Houde A, A systematic review/meta-analysis of primary research investigating swine, pork or pork products as a source of zoonotic hepatitis E virus. Epidemiol Infect. 2011;139:1127–44. 10.1017/S095026881100067721554782

[25] Crum-Cianflone N, Curry J, Drobeniuc J, Weintrob A, Landrum M, Ganesan A, ; Infectious Disease Clinical Research Program HIV Working Group. Hepatitis E virus infection in HIV-infected persons. Emerg Infect Dis. 2012;18:502–6. 10.3201/eid1803.11127822377220PMC3309581

# Research article: Laboratory-based Surveillance for Hepatitis E Virus Infection, United States, 2005–2012

**Authors:** Jan Drobeniuc, Tracy Greene-Montfort, Ngoc-Thao Le, Tonya R. Mixson-Hayden, Lilia Ganova-Raeva, Chen Dong, Ryan T. Novak, Umid M. Sharapov, Rania A. Tohme, Eyasu Teshale, Saleem Kamili, Chong-Gee Teo

**Affiliations:** Author affiliation: Centers for Disease Control and Prevention, Atlanta, GA, USA

## Medscape CME Activity

Medscape, LLC is pleased to provide online continuing medical education (CME) for this journal article, allowing clinicians the opportunity to earn CME credit. 

This activity has been planned and implemented in accordance with the Essential Areas and policies of the Accreditation Council for Continuing Medical Education through the joint sponsorship of Medscape, LLC and Emerging Infectious Diseases. Medscape, LLC is accredited by the ACCME to provide continuing medical education for physicians.

Medscape, LLC designates this Journal-based CME activity for a maximum of 1 *AMA PRA Category 1 Credit(s)*^TM^. Physicians should claim only the credit commensurate with the extent of their participation in the activity.

All other clinicians completing this activity will be issued a certificate of participation. To participate in this journal CME activity: (1) review the learning objectives and author disclosures; (2) study the education content; (3) take the post-test with a 70% minimum passing score and complete the evaluation at http://www.medscape.org/journal/eid; (4) view/print certificate.

**Release date: Release date: January 23, 2013; Expiration date: January 23, 2014**

## Learning Objectives

Upon completion of this activity, participants will be able to:

- Describe the percentage of hepatitis E among US patients with hepatitis who were seronegative for acute hepatitis A and B, including those who had and those who had not traveled abroad
- Compare characteristics of nontravelers vs travelers with hepatitis E
- Describe HEV genotypes among nontravelers vs travelers with hepatitis E.

## CME Editor

**Shannon O’Connor, ELS, **Technical Writer/Editor, Emerging Infectious Diseases. *Disclosure: Shannon O’Connor has disclosed no relevant financial relationships.*

## CME Author

**Laurie Barclay, MD, **freelance writer and reviewer, Medscape, LLC. *Disclosure: Laurie Barclay, MD, has disclosed no relevant financial relationships.*

## Authors

*Disclosures* ***: Jan Drobeniuc*** *;* ***Tracy Greene-Montfort*** *;* ***Le Ngoc-Thao*** *;* ***Tonya R. Mixson-Hayden*** *;* ***Lilia Ganova-Raeva*** *;* ***Chen Dong*** *;* ***Ryan T. Novak*** *;* ***Umid M. Sharapov*** *;* ***Rania A. Tohme*** *;* ***Eyasu Teshale*** *;* ***Saleem Kamili*** *; and* ***Chong-Gee Teo*** *have disclosed no relevant financial relationships.*
